# Night-shift work, circadian and melatonin pathway related genes and their interaction on breast cancer risk: evidence from a case-control study in Korean women

**DOI:** 10.1038/s41598-019-47480-2

**Published:** 2019-07-29

**Authors:** Thu-Thi Pham, Eun-Sook Lee, Sun-Young Kong, Jeongseon Kim, Sun-Young Kim, Jungnam Joo, Kyong-Ah Yoon, Boyoung Park

**Affiliations:** 1 National Cancer Center Graduate School of Cancer Science and Policy, Goyang-si, 10408 Republic of Korea; 2 Hospital, National Cancer Center, Goyang-si, 10408 Republic of Korea; 3 Research Institute, National Cancer Center, Goyang-si, 10408 Republic of Korea; 4 College of Veterinary Medicine, Konkuk University, Seoul, 05029 Republic of Korea; 5 Department of Medicine, Hanyang University College of Medicine, Seoul, 04763 Republic of Korea

**Keywords:** Preventive medicine, Breast cancer

## Abstract

Our purpose is to investigate the impact of circadian and melatonin pathway genes as well as their interactions with night-shift work (NSW) on breast cancer risk in Korean women. Information about NSW and other covariates was collected using a structured questionnaire and twenty-two polymorphisms in 11 genes were analyzed in a hospital-based case-control study with 941 cases of breast cancer and 959 controls. In analysis of the main effects of each single nucleotide polymorphisms(SNPs), variants in *CLOCK* rs11133373 was associated with breast cancer risk even after false discovery rate (FDR) correction (Odd Ratios (OR) = 1.38 (95% Confident Interval (CI) 1.14–1.69) in CG and CC compared to GG genotype. Analysis of *MTNR1A* rs2119882 demonstrated a decreased risk of breast cancer in CC compared to TT (p-FDR = 0.043). A correlation between NSW and breast cancer interaction was found in two loci. NSW increased risk of breast cancer in women who carried the heterozygote genotype of *CRY2* rs2292912 (OR = 1.98, 95% CI = 1.14–3.44) or carried at least one minor allele of *RORA* rs1482057 (OR = 2.20, 95% CI = 1.10–4.37). Our study results support a putative role for several loci of circadian genes and genes of melatonin biosynthesis and their interaction, and the gene interactions with NSW in the development of breast cancer.

## Introduction

Breast cancer is the leading cause of cancer related burden among women worldwide. Higher incidence of breast cancer in industrialized areas and increased trends observed elsewhere in the world^1^ have suggested that changes in lifestyle due to the industrialization of societies could be one of the causes for an increased incidence of breast cancer. Among them, light at night exposure in countries with a high level of light at night may contribute up to 30–50% of the increased risk for breast cancer^2^. In 2007, the International Agency for Research on Cancer (IARC) evaluated the carcinogenicity of night shift work (NSW) and classified it as a probable carcinogen to humans. The biological plausibility for increased risk of cancer with NSW is possibly related to the suppression of nocturnal melatonin secretion, the modification of estrogen utilization, or changes in the expression of clock genes^3^. Shift work suppresses the expression levels of core clock genes and also changes the behavior of melatonin pathway genes, leading to adverse effects on health status including an increased risk of breast cancer associated with circadian dysrhythmias^4^.

In contrast to this previous work, a recent meta-analysis of prospective epidemiological studies failed to show any effect of NSW on breast cancer development^5^. In addition, all of the studies regarding NSW and breast cancer conducted in Asia failed to show an association, suggesting that a different resistance to the exposure to light at night or a differing genetic distribution in an Asian population compared to other populations might account for the non-association observed in Asia^6,7^. Furthermore, circadian rhythms that substantially affect an individual’s shift work tolerance could be altered by factors such as gene-environment interactions^8^. Genetic polymorphisms related to the circadian genes or melatonin pathway genes have been considered as candidates for increased disease risk because of their relationships with circadian disruption and gene expression alterations. Clock genes have been found in breast epithelium, and clock gene expression has been shown to be influenced by both the breast cellular microenvironment and the nearby breast tissues. Disruption of the clock genes changes their expression and might increase risk of breast cancer^9^. Also, the increased incidence of breast cancer associated with the melatonin pathway genes is thought to be due to melatonin binding to and activation of MT1 and MT2, the melatonin receptors encoded by the *MTNR1A* and *MTNR1B* genes, triggering anti-oncostatic actions in human breast cancer cells^10^. Some previous epidemiological studies have investigated NSW and variations of circadian rhythm gene interactions on breast cancer risk; however, the results are controversial. Several studies have indicated an association between NSW, the single nucleotide polymorphisms (SNPs) in circadian genes, and breast cancer risk, but the results from some studies could not be replicated in other studies^11–15^. Epidemiological findings looking at the melatonin pathway genes have associated some loci in *MTNR1A* with a decreased breast cancer risk, however, all the loci in *AANAT* that encode enzymes involved in melatonin synthesis and in the control of day/night rhythm of melatonin production, did not demonstrate any association with breast cancer risk^16^.

Although several epidemiological studies have been conducted worldwide, up until now, no study focused in an Asian population has been conducted to elucidate the linkage of melatonin path genes, circadian genes and NSW with breast cancer. Considering that the frequency of variants in clock genes is reported to be significantly different between ethnicities^17^, the effects of SNPs and their interactions with NSW on breast cancer may be unique in Asian countries. Therefore, we aim to investigate the impact of variants in circadian and melatonin pathway genes and their interactions with NSW on breast cancer risk in a population of East-Asian women.

## Methods

### Study population

**Figure 1:**
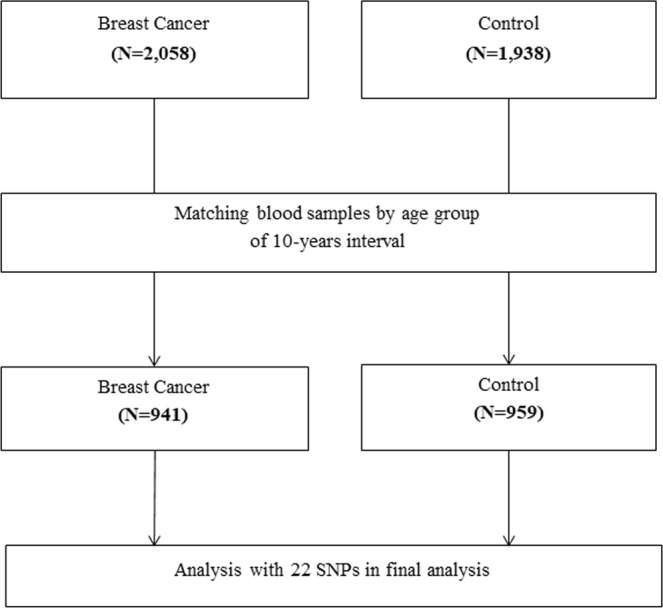
Flow chart of selecting study subjects.

A hospital-based case-control study was conducted in the National Cancer Center (NCC) of Korea to identify the factors associated with breast cancer risk. Cases were recruited from a population of women diagnosed with histologically confirmed breast cancer from 2012 to 2017 in the NCC. Controls were recruited from women who visited the NCC for health check-ups in the same period. Informed consent was collected for all cases and controls. The study protocol was reviewed and approved by the National Cancer Center Institutional Review Board (IRB number: NCCNCS13717). All procedures were performed in accordance with the Declaration of Helsinki 7^th^ version. The information on NSW experience, demographic characteristics, reproductive factors, and lifestyle behaviors were obtained using a structured questionnaire administered through face-to-face interviews. NSW was defined as ever having worked for night shifts regularly between 9:00 pm and 8:00am for at least 2 months in their lifetime. Among 2,058 cases and 1,938 controls with NSW information, after matching by 1:1 ratio according to the age groups of 10 year intervals and checking the availability of blood samples, 941 cases and 959 controls with both NSW information and blood samples were included (Fig. 1).

### Selection of polymorphisms and genotyping

Genomic DNA was extracted using peripheral blood leukocytes isolated from whole-blood samples obtained from the participants. We considered genetic variants in circadian and melatonin pathway genes based on articles published prior to January 2017 regarding the relationship of NSW, circadian genes or melatonin genes and risk of breast cancer. The SNPs which had been shown significantly associated with breast cancer in five studies were taken into account^11,13–15,18^. Among them, the SNPs with minor allele frequencies (MAFs) in East-Asian populations >0.15^19^ were included in this study. Information on 22 SNPs that are targeted during DNA sequencing, was obtained using high-throughput multiplex genotyping and Fluidigm BioMark™ System. The selected SNPs, chromosome locations and the protein function of each respective SNP are shown in Supplementary Table 1.

### Statistical analysis

The distribution of demographic characteristics and other covariates was described and tested by t-test and chi-square. For the 22 SNPs, the Hardy Weinberg equilibrium (HWE) was checked by performing an analysis which used 10,000 permutations in the controls groups to approximate P-values in the exact tests and SNPs with p value > 0.05 were included. We checked pairwise linkage equilibrium by calculating r^2^ for each of SNP pair and one SNP in high-correlated pairs was excluded. The frequency of genotypes and MAF for each SNP was checked in cases and controls separately. Chi-square test was computed using the permutation procedures to determine the difference among genotypes in case and control groups.

The association between each SNP and breast cancer risk was derived from unconditional multiple logistic regression models and presented as odd ratios (ORs) with 95% confidence intervals (CIs). When the number of cases or controls with minor allele was small (<5), exact logistic regression was used. All of the four models including codominant, dominant, recessive, and log-addictive were used to measure the strength of the association. The multiple correction adjusted P-values were obtained by 10,000 permutation testing and further checked by false discovery rate (FDR). This test is appropriate as recommended by Clarke *et al*. and has been applied by many authors^12,14,20^.

The gene-gene interaction was tested by adding each SNP pair term in the logistic regression model with the main effect. P-values for interaction were calculated by the log-likelihood ratio (LRT) test. These P-values were adjusted using FDR. The initial cut-off point of P-values was chosen at 0.20 as was done in a previous study^21^. SNPs with significant interactions were included in stratified analysis to illustrate the heterogeneity among genotypes of each SNP marker.

We tested the interaction between NSW and all of the SNPs in the circadian and melatonin pathway genes using the likelihood ratio test. The FDR adjusted method was also applied for p interaction values correction. We consider the initial cut-off point for P-value as 0.20. For the stratified analyses with NSW, NSW was divided into two classes (having ever/never exposed).

All analyses were adjusted for age at time of diagnosis or interview (as continuous variable), educational level, number of pregnancies, age at birth of first child, body mass index (BMI), age at menarche, alcohol consumption, smoking, use of female hormone treatment, and family history of breast cancer in first degree relatives. These variables were also included in the final logistic regression model to obtain adjusted odds ratios (ORs) for developing breast cancer associated with the distribution of polymorphisms alone (main effects analysis), and the polymorphisms and night work (stratified by presence or absence of night shifts). All statistical analyses were conducted by both SAS software version 9.4 and R version 3.5.0^22,23^.

### Ethical approval and informed consent

Informed consent was collected for all cases and controls. The study protocol was reviewed and approved by the National Cancer Center Institutional Review Board (IRB number: NCCNCS13717).

## Results

### Demographic characteristics of study population

The demographic characteristics of the study population are described in Table 1. Distribution of age in cases and controls are similar, and the mean ± standard deviation in cases and controls are 47.33 ± 8.64 and 48.30 ± 8.90, respectively. The distribution of number of pregnancies, age at birth of first child, BMI, age at menarche, tobacco smoking, alcohol drinking and family history of breast cancer were significantly different between the two groups (p < 0.05).

**Table 1:** Demographic characteristics of study population.

Characteristics	Controls (N = 941)		Cases (N = 959)		p-value^a^
	N	%	N	%	
Age group (years)					0.538
≤29	21	2.24	22	2.29	
30–39	102	10.84	124	12.93	
40–49	424	45.06	444	46.30	
50–59	293	31.14	288	30.03	
60–69	91	9.67	75	7.82	
≥70	10	1.06	6	0.63	
Number of pregnancies					<0.001
Nullipara	96	10.20	125	13.03	
1–2	279	29.65	327	34.10	
≥3	482	51.22	503	52.45	
Age at birth of first child (years)					<0.001
Nullipara	96	10.20	125	13.03	
<28	481	51.12	461	48.07	
≥28	252	26.78	341	35.56	
Education					0.902
<High school	359	38.15	372	38.79	
≥University	416	44.21	425	44.32	
BMI (kg/m^2^)					<0.001
<18.5	671	71.31	677	70.59	
≥18.5–24.9	53	5.63	39	4.07	
≥25	151	16.05	226	23.57	
Age at menarche (years)					<0.001
≤14	558	59.30	681	71.02	
>14	311	33.05	276	28.78	
Tobacco smoking					<0.001
Never	804	85.44	846	88.22	
Ever	78	8.29	113	11.78	
Alcohol drinking					<0.001
Never	476	50.58	681	71.01	
Ever	406	43.15	278	28.99	
Use of female hormone treatment in menopausal women^b^					0.197
Never	239	25.40	278	28.99	
Ever	81	8.61	74	7.72	
Family history of breast cancer in first degree relatives					<0.001
No	745	79.17	706	73.62	
Yes	129	13.71	231	24.09	
Menopausal status					<0.001
Premenopause	416	44.21	575	59.96	
Postmenopause	525	55.79	384	40.04	
Night shift work experience					0.497
Never	862	91.60	870	90.72	
Ever	79	8.40	89	9.28	
Duration of night shift work					0.459
Never	862	90.72	870	90.72	
<2 years	33	3.51	32	3.34	
≥2 years	43	4.57	56	5.84	
Unknown	3	0.32	1	0.10	
Estrogen receptor					
Negative	—		338	35.25	
Positive	—		586	61.11	
Unknown	—		35	3.65	
Progesteron receptor					
Negative	—		400	41.71	
Positive	—		525	54.74	
Unknown	—		34	3.55	
Human epidermal growth factor receptor 2					
Negative	—		697	72.68	
Positive	—		173	18.04	
Unknown	—		89	9.28	

N: number; BMI: body mass index (kg/m^2^); ^a^p value of chi-square test; ^b^in postmenopausal women.

### SNP information

Twenty-two SNPs in eleven genes (*AANAT, ARNTL, ARNTL1/BMAL1, ARNTL2/BMAL2, CLOCK, CRY2, CUL1, MTNR1A, NPAS2, RORA, TIMELESS*) from the circadian and melatonin signaling pathway with corresponding genotypes, frequencies of SNPs, less frequent alleles (LFA), MAF in study population, global LFA, global MAF, Hardy-Weinberg-derived P-values and P-value of chi-square test of genotypes are given in Supplementary Table 2. Two SNPs were excluded due to a high linkage distribution with another SNP. Thus 20 SNPs were considered in the final analysis.

### Individual SNPs and breast cancer association

All P-values from permutation test and FDR correction of four genetic models (codominant, dominant, recessive, and log-addictive) are presented in Supplementary Table 3. With the cut-off point for P-value at 0.05 using permutation test, three SNPs including rs11133373 and rs3749474 in the *CLOCK* gene, and rs2119882 in the *MTNR1A* gene have a statistically significant association with breast cancer (Supplementary Table 2 and Table 2). In addition, rs2291738 in *TIMELESS* gene showed a marginally significant association. However, after applying the FDR correction test, only two SNPs (rs11133373 and rs2119882) in the dominant model remained statistically significant (p = 0.043 and p = 0.025, respectively). The OR of rs11133373 CG/CC genotype was 1.38 (95% CI = 1.14–1.69) compared with GG and that of rs2119882 CT/CC genotype was 0.75 (95% CI = 0.61–0.91) compared with TT. When the effect of the interaction between individual SNPs and menopausal status on breast cancer was assessed, we found no significant association between SNPs and menopausal status.

**Table 2:** Odds ratios, 95% confidence intervals, permutation P-value, and FDR correction P-value as the association between each polymorphism and breast cancer risk.

Locus & genotypes	Model	Control^a^ (N = 941)		Cases^a^ (N = 959)		Crude Odd Ratios			Adjusted Odd Ratios^b^		
		N	%	N	%	OR (95% CI)	Perm p^c^	FDR p^d^	OR (95% CI)	Perm p^c^	FDR p^d^
CLOCK rs3749474											
T/T	Dominant	351	36.7	390	41.6	1	0.017	0.116	1	0.012	0.080
C/T-C/C		605	63.3	547	58.4	1.25 (1.04–1.51)			1.29 (1.06–1.57)		
T/T	Codominant	351	36.7	390	41.6	1	0.057	0.380	1	0.039	0.194
C/T		472	49.4	431	46	1.24 (1.02–1.51)			1.31 (1.06–1.62)		
C/C		133	13.9	116	12.4	1.29 (0.97–1.72)			1.22 (0.90–1.67)		
CLOCK rs11133373											
G/G	Dominant	362	38	421	44.9	1	0.001	0.022	1	0.001	0.025
C/G-C/C		591	62	516	55.1	1.36 (1.13–1.63)			1.38 (1.14–1.69)		
G/G	Codominant	362	38	421	44.9	1	0.005	0.092	1	0.005	0.095
C/G		475	49.8	419	44.7	1.35 (1.11–1.63)			1.41 (1.14–1.73)		
C/C		116	12.2	97	10.4	1.41 (1.04–1.92)			1.30 (0.94–1.79)		
G/G-C/G-C/C	Log-additive	953	50.4	937	49.6	1.24 (1.08–1.42)	0.002	0.046	1.22 (1.05–1.41)	0.008	0.159
MTNR1A rs2119882										941	
T/T	Dominant	380	40.3	318	34.2	1	0.008	0.080	1	0.004	0.043
C/T-C/C		564	59.7	611	65.8	0.78 (0.64–0.94)			0.75 (0.61–0.91)		
T/T	Codominant	380	40.3	318	34.2	1	0.029	0.292	1	0.014	0.142
C/T		424	44.9	462	49.7	0.77 (0.63–0.94)			0.73 (0.59–0.90)		
C/C		140	14.8	149	16	0.79 (0.60–1.04)			0.80 (0.59–1.07)		
TIMELESS rs2291738											
T/T-T/C	Recessive	855	89.5	812	86.8	1	0.058	0.527	1	0.013	0.264
C/C		100	10.5	124	13.2	0.76 (0.58–1.01)			0.69 (0.51–0.93)		
T/T	Codominant	395	41.4	392	41.9	1	0.109	0.545	1	0.038	0.194
T/C		460	48.2	420	44.9	1.09 (0.90–1.33)			1.07 (0.87–1.31)		
C/C		100	10.5	124	13.2	0.80 (0.59–1.08)			0.71 (0.52–0.98)		
T/T > T/C > C/C	Log-additive	955	50.5	936	49.5	0.95 (0.83–1.09)	0.478	0.831	0.91 (0.78–1.05)	0.179	0.593

OR: Odds ratios, 95% CI: 95% confidence intervals, Perm p: permutation P-value, FDR p: FDR correction P-value.

^a^Number of subjects in each group with successful genotyping data.

^b^Odd ratio after adjustment by age at time of diagnosis or interview, educational level, number of pregnancies, age at birth of first child, body mass index, age at menarche, alcohol consumption, smoking, use of female hormone treatment, and family history of breast cancer in first degree relatives.

^c^Adjust P-values for multiple comparisons were obtained by 10,000 permutation testing.

^d^Adjust P-values for multiple comparisons were obtained by false discovery rate method.

### Gene-gene interaction in the relationship with breast cancer risk

All of the permutation P-values and FDR correction P-values between pair of SNPs are plotted in Supplementary Fig. 1. Using a cut-off point of FDR correction P-value at 0.20, the significant interaction between two SNPs was found between *NPAS2* rs3820787 and *RORA* rs1482057, CUL1 rs758880 and *RORA* rs1482057, *CLOCK* rs11133373 and *CUL1* rs758880. The significantly increased risk of breast cancer according to the increasing number of *RORA* rs1482057 A allele was found in women who carry the GG genotype of *NPAS2* rs3820787 or the AA genotype of *CUL1* rs758880 (Table 3), otherwise with different genotypes, increment of *RORA* rs1482057 A genotype showed no association or decreased tendency for development of breast cancer. For those who carried the *CLOCK* rs11133373 CC genotype, increment of *CUL1* rs758880 A genotype increased the breast cancer risk (OR = 1.84 [95% CI = 1.13–3.00]), but in GG genotype, it showed decreased associations significantly.

**Table 3:** Odds ratios, 95% confidence intervals, and FDR correction P-value as the interaction effect of each pair of polymorphism on breast cancer risk.

Locus 1	Locus 2	Control^a^ (N = 941)		Case^a^ (N = 959)		Adj OR^b^	Permutation P interaction	FDR P interaction^c^
		N	%	N	%	(95% CI)		
NPAS2	RORA							
rs3820787	rs1482057						0.001	0.114
AA	One increment of A	296	31.46	290	30.24	0.82 (0.57–1.18)		
AG	One increment of A	458	48.67	466	48.59	0.93 (0.71–1.22)		
GG	One increment of A	178	18.92	193	20.13	2.52 (1.51–4.19)		
CUL1	RORA							
rs758880	rs1482057						0.002	0.129
GG	One increment of A	253	26.89	261	27.22	0.69 (0.48–1.01)		
GA	One increment of A	478	50.80	491	51.20	1.19 (0.91–1.56)		
AA	One increment of A	202	21.47	199	20.75	1.75 (1.08–2.81)		
CLOCK	CUL1							
rs11133373	rs758880						0.001	0.114
GG	One increment of A	421	44.74	360	37.54	0.80 (0.64–1.00)		
CG	One increment of A	416	44.21	474	49.43	0.99 (0.80–1.21)		
CC	One increment of A	97	10.31	116	12.10	1.84 (1.13–3.00)		

OR: Odds ratios, 95% CI: 95% confidence intervals, FDR: false discovery rate.

^a^Number of subjects in each group with successful genotyping data

^b^Odd ratio after adjustment by age at time of diagnosis or interview, educational level, number of pregnancies, age at birth of first child, body mass index, age at menarche, alcohol consumption, smoking, use of female hormone treatment, and family history of breast cancer in first degree relatives.

^c^P interaction after adjustment by false discovery rate method. P < 0.2 was considered noteworthy.

### NSW – SNPs interaction in the relationship with breast cancer risk

The permutation P-values and FDR correction P-values of NSW-gene interaction are plotted in Supplementary Fig. 2. Only two loci, rs2292912 and rs1482057, continued to have an interaction with NSW after FDR correction with P-value < 0.20. The interaction effects between NSW and these two SNPs on breast cancer risk are shown in Table 4. NSW increased risk of breast cancer in women who carried the CG genotype of rs2292912 in *CRY2* (OR = 2.03, 95% CI = 1.15–3.58), however for women who carried the GG or CC, ever engaging in NSW rather decreased the risk. For rs1482057 in *RORA*, women with at least an A allele who engaged ever in NSW had a higher risk of breast cancer (OR = 1.47, 95% CI = 1.17–1.84) but in women with the CC homozygote, ever engaging in NSW was not associated with breast cancer, and instead decreased the risk.

**Table 4:** Odds ratios, 95% confidence intervals, and FDR correction P-value as the interaction effect between night shift work and each polymorphism on breast cancer risk.

Locus * NSW	Control (N = 941)	Case (N = 959)	OR (95% CI)^a^	Permutation P interaction	FDR P interaction^b^
CRY2 rs2292912*NSW				0.0051	0.102
GG/no	95	86	ref.		
GG/yes	14	6	0.46 (0.16–1.34)		
CG/no	381	399	1.11 (0.79–1.57)		
CG/yes	26	51	**2.18 (1.18**–**4.04)**		
CC/no	379	378	1.10 (0.78–1.56)		
CC/yes	39	31	0.76 (0.42–1.39)		
CRY2 rs2292912*NSW					
GG/no	95	86	ref.		
GG/yes	14	6	0.51 (0.16–1.66)		
CG/no	381	399	ref.		
CG/yes	26	51	2.03 (1.15–3.58)		
CC/no	379	378	ref.		
CC/yes	39	31	0.68 (0.40–1.17)		
RORA rs1482057*NSW				0.0052	0.104
CC/no	627	631	ref.		
CC/yes	64	54	0.79 (0.53–1.19)		
CA-AA/no	230	235	0.98 (0.78–1.23)		
CA-AA/yes	15	35	**2.20 (1.10**–**4.37)**		
RORA rs1482057*NSW					
CC/no	627	631	ref.		
CC/yes	64	54	0.79 (0.53–1.19)		
CA-AA/no	230	235	ref.		
CA-AA/yes	15	35	**1.47 (1.17**–**1.84)**		

OR: Odds ratios, 95% CI: 95% confidence intervals, FDR: false discovery rate.

^a^Odd ratio after adjustment by age at time of diagnosis or interview, educational level, number of pregnancies, age at birth of first child, body mass index, age at menarche, alcohol consumption, smoking, use of female hormone treatment, and family history of breast cancer in first degree relatives.

^b^P interaction after adjustment by false discovery rate method. P < 0.2 was considered noteworthy.

## Discussion

In this study, we investigated the associations between genes of circadian regulation, melatonin pathway and breast cancer risk. Our study results support a putative role of several loci in circadian genes (*CLOCK, TIMELESS*) and genes of melatonin biosynthesis (*MTNR1A*) in the development of breast cancer. In addition, putative gene interactions between NPAS2, CUL1 and RORA, CLOCK and CUL1 is observed to have correlation in the development of breast cancer. The effect of NSW appears to be modified by variants of CRY2 and RORA genes.

Among twenty SNPs in circadian and melatonin pathway genes^11–14,20^, around 2/3 of the examined SNPs in our study population had different allele distributions with different minor alleles compared to published Western populations, supporting the possibility that the different ethnic groups would also differ as to the effect of clock gene SNPs in breast cancer^17^. Previous studies reported significant associations between variants of different loci in the *CLOCK* gene and breast cancer. In a previous study, a decreased risk of breast cancer was recorded in women who carried a TT genotype of rs3749474, with an OR of 0.64 (95% CI 0.45–0.92) which was comparable with our results but no association between rs11133373 and breast cancer risk was found^13^. However, in this study an association between *CLOCK* rs11133373 and breast cancer risk in log-additive models when C allele were considered as a reference (OR = 0.82, 95% CI = 0.71–0.94) is observed and it is similar with another previous result^15^. *CLOCK* rs2035691 is found to have no association with breast cancer risk in both Asian and European women^20^ and rs10462028 has no association with breast cancer risk^18^. Another finding indicated that breast cancer risk in postmenopausal women is associated with rs11932595 in *CLOCK*^11^. A possible explanation for the association between variants in the *CLOCK* gene and breast cancer risk would be that hypermethylation of the *CLOCK* promoter reduces the risk of breast cancer^24^. We checked and compared less frequent alleles and MAF of these significant SNPs in our study, MAF in Chinese and Japanese populations and confirmed no difference, as may be predicted in comparing results with other East-Asian populations^25^.

Other genes were also indicated in published studies for the association with breast cancer risk. A significant association between *TIMELESS* rs2291738 and breast cancer was observed with effect modification of hormone receptor status of breast cancer. The GG genotype of rs2291738 decreased hormone receptor positive breast cancer but not overall breast cancer^26^. Comparable with previous results, our study shows the CC genotype of rs2291738 decreased overall breast cancer risk. For other SNPs in *TIMELESS* genes, rs774036 was not associated with breast cancer risk in both European and Asian women^20^. In terms of melatonin pathway genes, *MTNR1B* rs10765576 showed inconsistent results in previous studies – reduced risk of breast cancer among those who carried AA allele^16^ or no association^18^, and *MTNR1A* rs7665392 showed different effects on breast cancer by menopausal status^16^. Other SNPs in *MTNR1A* or *MTNR1B* did not show association with breast cancer^16^. However, our study demonstrates that *MTNR1A* rs2119882 CT and TT genotype decreased breast cancer risk. One study considered rs2119882 but then excluded it due to HWE^14^.

In terms of gene-gene interactions of circadian and melatonin pathway genes on the relationship with breast cancer, two-way interactions between *ARNTL/MTNR1B*, *MTNR1B/NPAS2* and three-way interactions with *MTNR1B/NPAS2/ARNTL* was observed in a previous study^18^. In our study, interactions between combinations of *RORA* and *NPAS2, RORA* and *CUL1*, and *CUL1* and *CLOCK* are observed. Based on our findings, the potential role of *CLOCK* in breast cancer etiology seems plausible referring to both a main effect of individual SNP and gene-gene effects on breast cancer. Furthermore, loci in *RORA* play an important part in the interaction with other SNPs.

In our study, SNPs having significant main effects on breast cancer in this study (Table 2) did not show an interaction effect with NSW on breast cancer. However, rs2292912 in *CRY2* and rs1482057 in *RORA* are observed to have possible interactions with shift work. A previous study showed interaction effects of NSW and various genes including *CLOCK, CRY2, RORB, AANAT, MTNR1A* on breast cancer^14^. In addition, women with the *NPAS2* Ala394Thr (rs23051560) Thr/Thr variation showed increased breast cancer risk by about three times when they engaged in NSW for 24 months or more but did not show association in other genotypes^13^. However, two other previous studies did not find interaction effects between any of the examined genes and NSW on breast cancer risk^11,20^. Each SNP which showed interaction with NSW in only one study was not replicated in other previous studies. The interaction between *CRY2* and NSW is observed in this study and in Zienolddiny *et al*. despite of different SNP in *CRY2* gene^14^. Despite differences in SNP, according to Zienolddiny *et al*., genotype of SNPs in *CRY2*, the effect of NSW was different, especially heterozygote of rs11038689 as our study results^14^. The variants in *CRY2* may affect breast cancer through the hormone signaling pathway^27^. Considering the effect of NSW in the modification of estrogen utilization^3^, the effect of interaction between polymorphisms of *CRY2* and NSW on breast cancer can be explained. In addition, Zienolddiny *et al*. reported that not only *CRY2*, but also rs3903529 in *RORB* interacts with NSW and effects breast cancer risk with different direction of effect of NSW in those carrying heterozygote genotype^14^. Our results as an interaction between exactly same SNP and NSW have not been replicated in previously published results from Western countries. Differences in the genetic structure between the Asian and the Western population might could be the reason behind this variation^28,29^. Thus, investigation of these interactions in larger populations is needed, especially in the Asian population. Due to the small number of the subjects who had experience of NSW and each genotype, the most results for the interaction effect between night shift work and each SNP on breast cancer risk showed wide CIs, yielding inconclusive results, possibly caused by limited statistical power. However, our study is the first study conducted using an Asian population to examine the association of circadian genes, melatonin pathway genes and breast cancer, as well as genes-NSW interaction in relationship to the etiology of breast cancer. This is a carefully designed case–control study with detailed information in covariates. The sample size of our study is round 2,000, which is similar with previous studies^13,18,20^.

The study may have a number of limitations. In defining NSW, we did not take into account the detailed night-shift characteristics such as shift duration or rotation cycles. We just considered the experience of NSW due to the limited number of subjects who were night-shift workers [89 (9.3%) and 79 (8.4%) of 959 breast cancer patients and 941 controls, respectively]. Thus, stratification analysis according to detailed NSW information such as duration of NSW or the number of consecutive NSW shifts could not be conducted. The Korean 3rd Working Condition Survey reported that 7.5% of female workers were involved in night-shift work^30^, which is comparable to the percentage of night shift workers in our study. In addition, the number of women with experience of NSW in this study was comparable to that of previous population based case-control studies, which included approximately 100 patients and 100 controls with experience of NSW^11,18^. We selected a limited number of variants in circadian and melatonin pathway genes used in the previous studies, and, to cover all the SNPs in these genes to determine relevant makers in an East-Asian population, a much larger study is warranted. The breast cancer patients and controls were recruited from a single cancer center. Therefore, the external validity may be limited although whether the variants met HWE is considered in the analysis. Recall-bias could affect the result in the measurement of NSW and other covariates. In addition, although the sample size was adequate to identify the SNPs with a frequency of MAF of around 0.15 and OR of 1.4, for SNPs with smaller frequency or smaller ORs, the statistical power would not be enough. In addition, considering the small number of subjects who had engaged NSW, the power to detect interaction effect between NSW and relevant SNPs on breast cancer risk was reduced.

Our hospital based case-control data supported the possible role of several variants in genes of circadian regulation and the melatonin pathway associated with breast cancer risk, both with an individual effect and an interaction of genes effect. In addition, *CRY2* and *RORA* genes were denoted for the effect on breast cancer though the interaction with other genes or with an environmental factor (NSW). Further study should be conducted in large populations to replicate this finding and investigate this association in a stratified analysis based on menopausal status and varying levels of shift work exposure.

## Supplementary information

Supplementary information

## Data Availability

Data is available on request to the authors.
