# Supplementary material for: Night-shift work, circadian and melatonin pathway related genes and their interaction on breast cancer risk: evidence from a case-control study in Korean women
Source: Sci Rep. 2019 Jul 29;9:10982. doi: 10.1038/s41598-019-47480-2 (PMC6662707; doi:10.1038/s41598-019-47480-2)
Supplement: Supplementary file 1 — Supplementary information [file 41598_2019_47480_MOESM1_ESM.pdf]

Title: Night-shift work, circadian and melatonin pathway related genes and their interaction on breast cancer risk: evidence from a case-control study in Korean women

Authors and affiliations:

Thu-Thi Pham, Pharm, MPH [1] (thuphamhup@gmail.com)

Eun-Sook Lee, MD, PhD[1,2,3] (eslee@ncc.re.kr)

Sun-Young Kong, MD, PhD[1,2,3] (ksy@ncc.re.kr)

Jeongseon Kim, PhD[1,3] (jskim@ncc.re.kr)

Sun-Young Kim, PhD[1] (sykim@ncc.re.kr)

Jungnam Joo, PhD[3] (jooj@ncc.re.kr)

Kyong-Ah Yoon, PhD[3,4] (kayoon@ncc.re.kr)

Boyoung Park, MD, PhD[5] (hayejine@hanmail.net)

Supplementary Table 1: Twenty-two candidate SNPs, chromosome locations and the protein function of each respective SNP

No.	GENE	Candidate SNP	Chromosome	Protein function	Note
1	AANAT	rs8150	chr17	the metabolism of melatonin	
2	ARNTL	rs3816358	chr11	circadian regulation	
3	ARNTL	rs3816360	chr11	circadian regulation	
4	ARNTL1/BMAL1	rs2278749	chr11	circadian regulation	
5	ARNTL1/BMAL1	rs2290035	chr11	circadian regulation	
6	ARNTL2/BMAL2	rs2306074	chr12	circadian regulation	
7	CLOCK	rs10462028	chr4	circadian regulation	
8	CLOCK	rs11133373	chr4	circadian regulation	
9	CLOCK	rs3749474	chr4	circadian regulation	
10	CRY2	rs2292912	chr11	circadian regulation	
11	CRY2	rs7951225[a]	chr11	circadian regulation	LD
12	CUL1	rs243482[a]	ch7	circadian regulation	LD
13	CUL1	rs758880	chr7	circadian regulation	
14	MTNR1A	rs2119882	chr4	the metabolism of melatonin	
15	NPAS2	rs12712085	chr2	circadian regulation	
16	NPAS2	rs17024926	chr2	circadian regulation	
17	NPAS2	rs2305160	chr2	circadian regulation	
18	NPAS2	rs3820787	chr2	circadian regulation	
19	RORA	rs1482057	chr15	Both circadian and melatonin regulation	
20	RORA	rs2279295	chr15	Both circadian and melatonin regulation	
21	RORA	rs7164773	chr15	Both circadian and melatonin regulation	
22	TIMELESS	rs2291738	chr12	circadian regulation	

[a] SNPs were excluded from analysis due to r^2 of pairwise LD >0.8. LD: linkage disequilibrium.

Supplementary Table 2: Genetic characteristics of study population

Locus	Genotype	Controls (N=941)		Case [a] (N=959)		Minor allele	MAF	Global Minor allele	GMAF	P[a] for HWE	P[b] chisq for genotype
		N	%	N	%						
**rs12712085**	A/A	175	18.60	210	21.90	A	0.47	A	0.33	0.221	0.264
	A/G	476	50.58	476	49.64						
	G/G	275	29.22	271	28.26						
**rs1482057**	A/A	19	2.02	25	2.61	A	0.15	A	0.18	0.917	0.505
	A/C	226	24.02	245	25.55						
	C/C	691	73.43	685	71.43						
**rs2278749**	T/T	32	3.40	38	3.96	T	0.18	T	0.12	0.599	0.756
	C/T	269	28.59	266	27.74						
	C/C	634	67.38	652	67.99						
**rs2279295**	G/G	58	6.16	62	6.47	G	0.24	G	0.28	0.278	0.922
	A/G	326	34.64	339	35.35						
	A/A	555	58.98	558	58.19						
**rs2292912**	G/G	109	11.58	92	9.59	G	0.33	G	0.5	0.514	0.171
	C/G	407	43.25	450	46.92						
	C/C	418	44.42	409	42.65						
**rs3749474**	C/C	116	12.33	133	13.87	C	0.39	T	0.38	0.855	0.085
	C/T	431	45.80	472	49.22						
	T/T	390	41.45	351	36.60						
**rs3816358**	A/A	27	2.87	19	1.98	A	0.15	A	0.11	0.493	0.462
	A/C	247	26.25	256	26.69						
	C/C	663	70.46	682	71.12						
**rs3820787**	G/G	179	19.02	193	20.13	G	0.45	G	0.37	0.991	0.788
	A/G	460	48.88	469	48.91						
	A/A	296	31.46	291	30.34						
**rs7164773**	C/C	362	38.47	372	38.79	T	0.38	T	0.48	0.361	0.388
	C/T	450	47.82	441	45.99						
	T/T	123	13.07	146	15.22						
**rs758880**	A/A	202	21.47	201	20.96	A	0.47	A	0.43	0.412	0.961
	A/G	479	50.90	492	51.30						
	G/G	255	27.10	262	27.32						
**rs7951225**	A/A	419	44.53	412	42.96	T	0.33	T	0.5	0.547	0.205
	A/T	409	43.46	451	47.03						
	T/T	109	11.58	93	9.70						
**rs8150**	C/C	389	41.34	391	40.77	G	0.37	G	0.38	0.939	0.636
	C/G	429	45.59	431	44.94						
	G/G	117	12.43	134	13.97						
**rs2290035**	A/A	61	6.48	57	5.94	A	0.26	A	0.43	0.908	0.762
	A/T	353	37.51	375	39.10						
	T/T	521	55.37	524	54.64						
**rs3816360**	C/C	82	8.71	106	11.05	C	0.32	T	0.5	0.103	0.123

	C/T	425	45.16	400	41.71						
	T/T	428	45.48	453	47.24						
**rs2306074**	C/C	338	35.92	320	33.37	T	0.41	T	0.44	0.204	0.282
	C/T	463	49.20	480	50.05						
	T/T	133	14.13	157	16.37						
**rs11133373**	C/C	97	10.31	116	12.10	C	0.37	C	0.31	0.629	0.008
	C/G	419	44.53	475	49.53						
	G/G	421	44.74	362	37.75						
**rs10462028**	A/A	6	0.64	10	1.04	A	0.10	A	0.25	0.369	0.514
	A/G	165	17.53	177	18.46						
	G/G	767	81.51	770	80.29						
**rs17024926**	C/C	237	25.19	246	25.65	C	0.50	C	0.35	0.552	0.535
	C/T	478	50.80	466	48.59						
	T/T	223	23.70	246	25.65						
**rs2305160**	A/A	35	3.72	39	4.07	A	0.21	A	0.2	0.635	0.752
	A/G	302	32.09	325	33.89						
	G/G	590	62.70	594	61.94						
**rs243482**	A/A	258	27.42	260	27.11	C	0.47	C	0.44	0.690	0.852
	A/C	471	50.05	494	51.51						
	C/C	204	21.68	201	20.96						
**rs2119882**	C/C	149	15.83	140	14.60	C	0.37	C	0.47	0.382	0.027
	C/T	462	49.10	424	44.21						
	T/T	318	33.79	380	39.62						
**rs2291738**	C/C	124	13.18	100	10.43	C	0.35	C	0.33	0.493	0.119
	C/T	420	44.63	460	47.97						
	T/T	392	41.66	395	41.19						

MAF: Minor Allele Frequency; GMAF: Global Minor Allele Frequency; HWE: Hardy–Weinberg Equilibrium.

[a] Exact p-values of Hardy Weinberg equilibrium (HWE) were checked by performing analysis which used 10,000 permutations in the control group.

[b] P values of the chi-square statistic for genotypes. Chi-square test was computed using the permutation procedures to determine the difference among genotypes in case and control groups.

Supplementary Table 3: P values of logistic regression of each SNP on breast cancer in perm (permutation) test and FDR correction. The factors adjusted in regression model: age at time of diagnosis or interview, educational level, number of pregnancies, age at birth of first child, body mass index, age at menarche, alcohol consumption, smoking, use of female hormone treatment, and family history of breast cancer in first degree relatives.

	Codominant model		Dominant model		Recessive model		Log-additive model	
	perm test	fdr correction	perm test	fdr correction	perm test	fdr correction	perm test	fdr correction
rs12712085	0.212	0.706	0.518	0.837	0.078	0.714	0.151	0.593
rs1482057	0.494	0.759	0.544	0.837	0.254	0.714	0.383	0.696
rs2278749	0.555	0.777	0.524	0.837	0.507	0.832	0.753	0.837
rs2279295	0.985	0.985	0.977	0.977	0.880	0.926	0.970	0.970
rs2292912	0.333	0.759	0.593	0.847	0.248	0.714	0.890	0.936
rs3749474	0.039	0.194	0.012	0.080	0.707	0.832	0.046	0.304
rs3816358	0.343	0.759	0.495	0.837	0.155	0.714	0.301	0.670
rs3820787	0.752	0.885	0.471	0.837	0.979	0.979	0.626	0.837
rs7164773	0.583	0.777	0.849	0.915	0.366	0.732	0.743	0.837
rs758880	0.896	0.965	0.870	0.915	0.640	0.832	0.703	0.837
rs8150	0.391	0.759	0.467	0.837	0.181	0.714	0.235	0.593
rs2290035	0.917	0.965	0.708	0.885	0.779	0.865	0.678	0.837
rs3816360	0.140	0.561	0.219	0.837	0.268	0.714	0.662	0.837
rs2306074	0.478	0.759	0.376	0.837	0.285	0.714	0.237	0.593
rs11133373	0.005	0.095	0.001	0.025	0.610	0.832	0.008	0.159
rs10462028	0.621	0.777	0.392	0.837	0.537	0.832	0.351	0.696
rs17024926	0.461	0.759	0.673	0.885	0.336	0.732	0.744	0.837
rs2305160	0.285	0.759	0.114	0.572	0.587	0.832	0.124	0.593
rs2119882	0.014	0.142	0.004	0.043	0.705	0.832	0.029	0.290
rs2291738	0.038	0.194	0.854	0.915	0.013	0.264	0.179	0.593

perm test: P of permutation test; fdr correction: P of false discovery rate test.

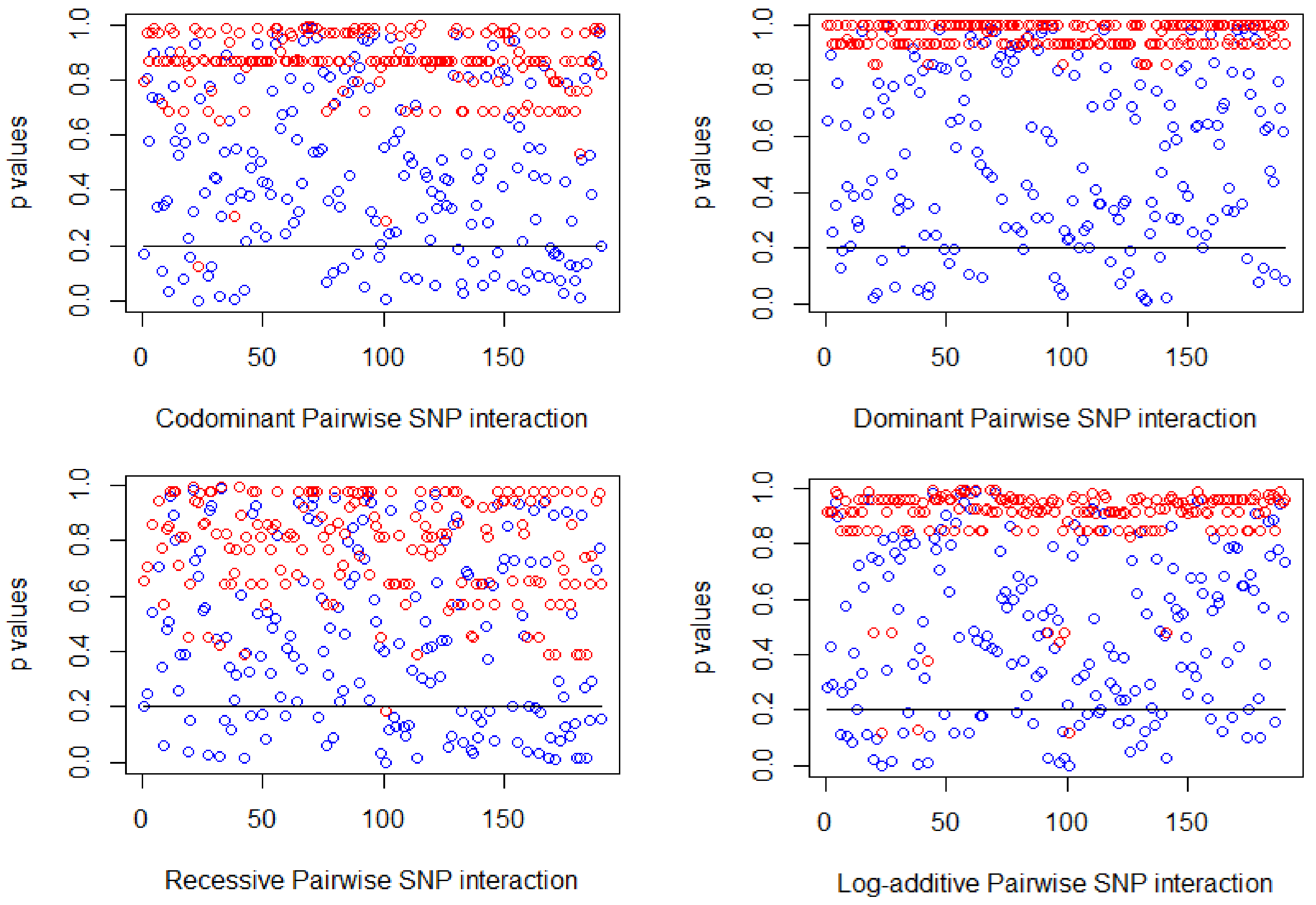
p values
1.0
0.8
0.6
0.4
0.2
0.0
0
50
100
150
Codominant Pairwise SNP interaction
Dominant Pairwise SNP interaction
Recessive Pairwise SNP interaction
Log-additive Pairwise SNP interaction

Supplementary Figure 1: P values of gene-gene interaction before and after FDR adjustment among 4 genetic models in logistic regression model with adjusted factors (age at time of diagnosis or interview, educational level, number of pregnancies, age at birth of first child, body mass index (BMI), age at menarche, alcohol consumption, smoking, use of female hormone treatment, and family history of breast cancer in first degree relatives). Blue plots showed p values of permutation tests while red plots showed p values of FDR correction test.

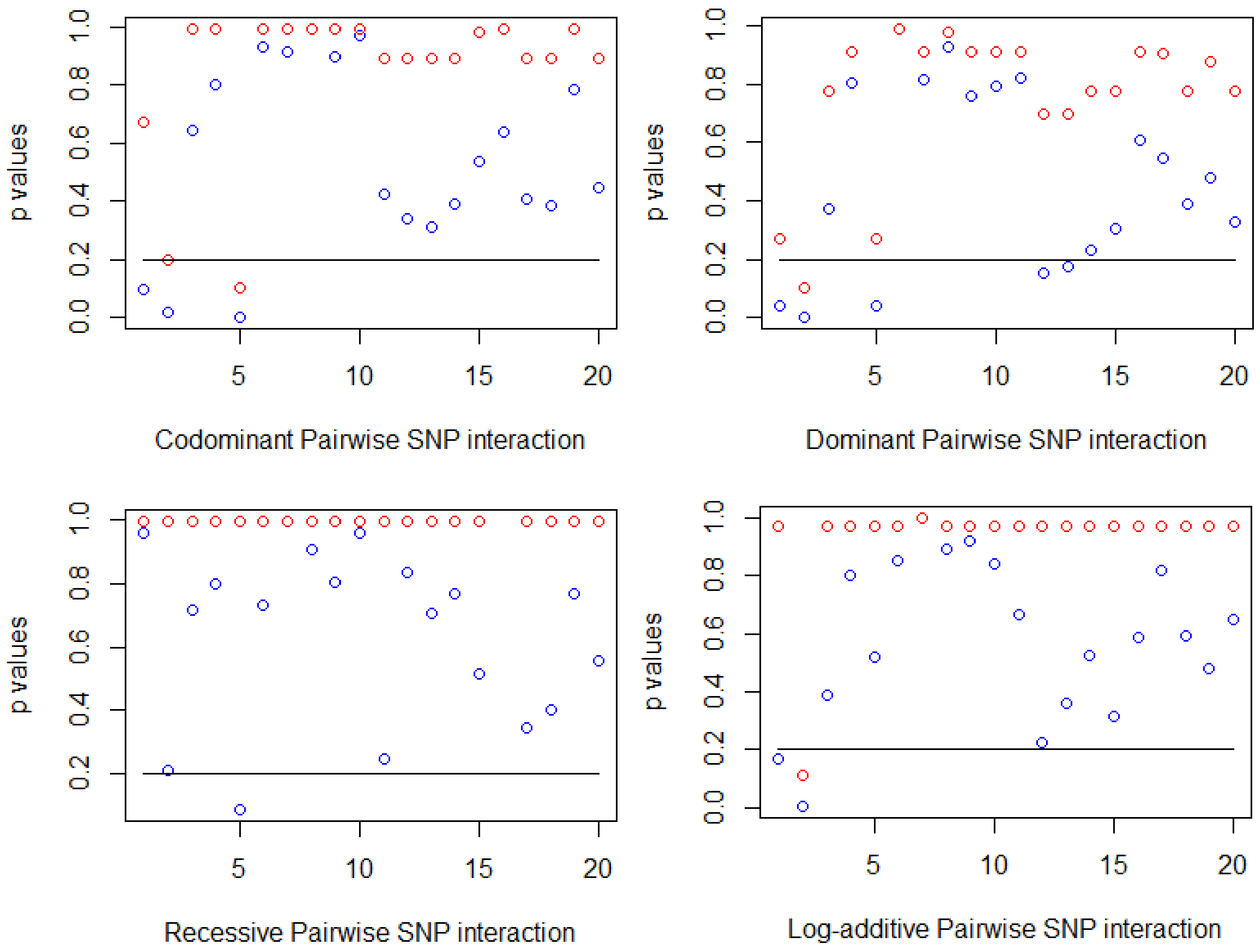
p values
0.0
0.2
0.4
0.6
0.8
1.0
5
10
15
20
Codominant Pairwise SNP interaction
Dominant Pairwise SNP interaction
Recessive Pairwise SNP interaction
Log-additive Pairwise SNP interaction

Supplementary Figure 2: Plot of p of NSW – SNPs interaction before and after FDR adjustment among 4 genetic models in logistic regression model with adjusted factors (age at time of diagnosis or interview, educational level, number of pregnancies, age at birth of first child, body mass index (BMI), age at menarche, alcohol consumption, smoking, use of female hormone treatment, and family history of breast cancer in first degree relatives). Blue plots showed p values of permutation tests while red plots showed p values of FDR correction test.
